# Relationship of hypoxia to metastatic ability in rodent tumours

**DOI:** 10.1054/bjoc.2001.1743

**Published:** 2001-05

**Authors:** K De Jaeger, M-C Kavanagh, R P Hill

**Affiliations:** Experimental Therapeutics Division,Research Department and Department of Radiation Oncology,Ontario Cancer Institute/Princess Margaret Hospital and Departments of Medical Biophysicsand Radiation Oncology,University of Toronto, 610 University Avenue, Toronto, Ontario, Canada M5G 2M9

**Keywords:** hypoxia, metastasis, rodent tumours, polarographic oxygen sensors

## Abstract

The relationship between tumour oxygenation in vivo and metastatic potential was investigated in 2 rodent tumour models, KHT-C fibrosarcoma and SCC-VII squamous cell carcinoma. The oxygen status in these rodent tumours transplanted intramuscularly in syngeneic mice was measured using the Eppendorf pO _2_ Histograph. The results indicate a considerable heterogeneity in oxygenation between individual tumours within each tumour cell line. At different tumour sizes, animals were killed and lung lobes were examined for macroscopic and microscopic lung metastases. In the KHT-C tumours, a significant increase in early pulmonary metastasis formation was observed in mice with hypoxic primary tumours. Hypoxic SCC-VII tumours did not give rise to enhanced lung metastasis formation despite oxygenation in a range similar to the KHT-C tumours. However, the overall metastasis incidence in the SCC-VII model was very low. The results obtained in the KHT-C model, which show that hypoxic tumours are more likely to metastasize, are in agreement with recent clinical data suggesting that a hypoxic environment might be implicated in metastatic ability of human tumours. © 2001 Cancer Research Campaign http://www.bjcancer.com


*British Journal of Cancer* (2001) **84**(9), 1280–1285
© 2001 Cancer Research Campaign
doi: 10.1054/ bjoc.2001.1743, available online at http://www.idealibrary.com on IDEAL®

### Relationship of hypoxia to metastatic ability in rodent tumours

**K De Jaeger[1,2,3,4], M-C Kavanagh[1,3] and RP Hill[1,3,4]**

Experimental Therapeutics Division[1], Research Department and Department of Radiation Oncology[2], Ontario Cancer Institute/Princess Margaret Hospital and Departments of Medical Biophysics[3] and Radiation Oncology[4], University of Toronto, 610 University Avenue, Toronto, Ontario, Canada M5G 2M9

**Summary** The relationship between tumour oxygenation in vivo and metastatic potential was investigated in 2 rodent tumour models, KHT-C fibrosarcoma and SCC-VII squamous cell carcinoma. The oxygen status in these rodent tumours transplanted intramuscularly in syngeneic mice was measured using the Eppendorf $pO_2$ Histograph. The results indicate a considerable heterogeneity in oxygenation between individual tumours within each tumour cell line. At different tumour sizes, animals were killed and lung lobes were examined for macroscopic and microscopic lung metastases. In the KHT-C tumours, a significant increase in early pulmonary metastasis formation was observed in mice with hypoxic primary tumours. Hypoxic SCC-VII tumours did not give rise to enhanced lung metastasis formation despite oxygenation in a range similar to the KHT-C tumours. However, the overall metastasis incidence in the SCC-VII model was very low. The results obtained in the KHT-C model, which show that hypoxic tumours are more likely to metastasize, are in agreement with recent clinical data suggesting that a hypoxic environment might be implicated in metastatic ability of human tumours. © 2001 Cancer Research Campaign http://www.bjcancer.com

**Keywords**: hypoxia; metastasis; rodent tumours; polarographic oxygen sensors

It is well-documented that most human and rodent solid tumours contain a significant proportion of hypoxic cells (Rockwell et al, 1986; Rockwell and Moulder, 1990). From radiobiology studies, hypoxia is known to render tumour cells resistant to ionizing irradiation (Bristow and Hill, 1998). Methods to detect hypoxia might allow identification of patients with radioresistant tumours who would benefit from selective, hypoxia-targeting treatment strategies. Presently, the determination of oxygen concentration with polarographic electrodes is the only method of measuring hypoxia that has been extensively studied in patients. Decreased tumour oxygenation, as measured with polarographic electrodes, has been reported to be a predictor of poor local response following radiotherapy in cervix cancer (Höckel et al, 1993; Fyles et al, 1998; Sundfør et al, 1998; Knocke et al, 1999; Lyng et al, 2000) head and neck cancer (Gatenby et al, 1988; Nordsmark et al, 1996; Brizel et al, 1997) and soft tissue sarcoma (Brizel et al, 1996). Recently, clinical data have emerged suggesting that hypoxia adversely affects locoregional control of cervical cancer, irrespective whether the initial treatment modality is radiotherapy or surgery (Höckel et al, 1996). For soft tissue sarcoma, poorer oxygenation has also been linked to increased likelihood of developing distant metastasis (Brizel et al, 1996). Thus, in addition to radioresistance, hypoxia may be implicated in local tumour aggressiveness and distant progression.

Our lab has previously demonstrated in a murine model that metastasis formation by rodent tumour cells can be increased by exposure to hypoxia (Young et al, 1988). When murine fibrosarcoma cells were exposed to hypoxia in vitro they acquired a transient, enhanced ability to form experimental metastases. We hypothesized that a hypoxic environment induces genomic instability, possibly through gene amplification. Other investigators have reported hypoxia-mediated increased mutation frequencies in tumours (Reynolds et al, 1996) and selection of cells deficient in genes of normal regulatory pathways (Graeber et al, 1996; Kim et al, 1997). In addition, it is well recognized that hypoxic stimuli can alter gene expression by up-regulating specific transcription factors and, at a post-transcriptional level, by increasing messenger RNA stability (Ikeda et al, 1995; Dachs and Stratford, 1996; Iyer et al, 1998; Sutherland, 1998; Dachs and Tozer, 2000; Semenza, 2000). Intensive investigations are ongoing to elucidate the complex mechanisms underlying these epigenetic/genetic interactions.

In the previous study by Young et al (1988) metastasis formation was examined with rodent tumour cells after exposure to hypoxia in vitro and intravenous injection of tumour cells back into the animal. The availability of polarographic electrodes allowing direct measurements of tumour oxygenation stimulated us to re-address the question of whether hypoxia affects distant metastasis formation using a spontaneous metastasis model. In the current study we performed direct measurements of oxygenation in individual primary tumours in vivo and investigated their ability to form metastases in the lungs.

### MATERIALS AND METHODS

### Mice and tumour cell lines

Experiments were carried out with 2 murine tumour cell lines, KHT-C fibrosarcoma and SCC-VII squamous cell carcinoma. Their origin has been described previously (Bristow et al, 1990). Both cell lines were maintained in the present lab by alternate in vitro and in vivo passage. In vitro passage was done in plastic flasks, growing cells as monolayers in α-minimal essential

*Received 4 September 2000*
*Revised 18 January 2001*
*Accepted 22 January 2001*

*Correspondence to:* RP Hill medium (Gibco BRL, Burlington, Ontario) supplemented with 10% fetal bovine serum (FBS, Wisent, Quebec). Cells were removed from the monolayer while in exponential growth with 0.05% trypsin for 5 minutes at 37˚C. Tumour cells were used for experiments between their 2–4th passage in vitro and established in syngeneic 8–12-week-old C3H/HeJ male mice (The Jackson Laboratory, Bar Harbor, Maine). Approximately $2.5 \times 10^5$ cells, suspended in 30–50 µl growth medium were injected into the left gastrocnemius muscle. Tumour growth was followed by external measurement of the diameter of the tumour-bearing leg. All animals were selected for oxygen measurements when this diameter reached a size of 9 (± 0.5) mm (corresponding tumour weight 0.3–0.4 g). This generally occurred 8 days after injection. Animals were housed at the Ontario Cancer Institute animal colony and had access to food and water ad libitum. Experiments were performed according to the regulations provided by the Canadian Council on Animal Care.

### Tumour oxygenation measurements

Direct oxygen measurements were made in individual tumours using a polarographic oxygen electrode (Eppendorf $pO_2$ Histograph, Kimoc 6650, Hamburg, Germany) as reported previously (Kavanagh et al, 1996, 1999). Calibrations were performed according to the manufacturer's recommendations. All $pO_2$ measurements were made approximately 15 minutes after induction of anaesthesia with intraperitoneally injected Ketalean (ketamine hydrochloride, 50 mg $kg^{-1}$) (M.T.C. Pharmaceuticals, Cambridge, Ontario) and Rompun (xylazine, 5 mg $kg^{-1}$) (Bayvet Division, Chemagro Limited, Etobicoke, Ontario). Anaesthetized mice were positioned on a heating pad. Core temperature was monitored and kept at 37 ± 2˚C. In each tumour, 8–12 measurements were made along each of 6 parallel tracks resulting in a total of 48–72 $pO_2$ values per tumour. The $pO_2$ data for each tumour were corrected for tumour temperature, which was measured at one point similar in position to an Eppendorf track, using a 25 gauge needle thermocouple probe (Model #2300A, Fluke Electronics Canada Inc, Mississauga, Ontario). Oxygen measurements were performed in a total of 103 KHT-C tumours and 67 SCC-VII tumours at a tumour weight of 0.3–0.4 g. This was done in several experiments. In each experiment a number of mice were randomly allocated for cervical dislocation immediately after $pO_2$ measurements. Mice that were not sacrificed following oxygen measurements were monitored during recovery and kept under close surveillance until the tumour bearing leg reached a diameter of 15 (± 0.5) mm (corresponding tumour weight 1.8–1.9 g).

### Metastasis assessment

As lungs are the primary site of metastasis formation from leg tumours for both KHT-C and SCC-VII cells, the development of lung metastases was assessed.

After oxygen measurements at a tumour weight of 0.3–0.4 g, a total of 86 tumours (40 KHT-C and 46 SCC-VII) randomly selected from the group KHT-C and SCC-VII tumours were grown until the tumour-bearing leg had reached a tumour weight of 1.6–1.9 g. At this tumour size, the animals were killed by cervical dislocation, their lungs were removed, briefly washed with distilled water, cleaned of extraneous tissue, fixed in Bouin's solution overnight (BDH Inc, Toronto, Ontario) and stored in buffered formalin 10% (BDH Inc, Toronto, Ontario) until they were counted.

A total of 84 animals (63 bearing KHT-C and 21 SCC-VII tumours) was sacrificed immediately after oxygen measurements at a tumour weight of 0.3–0.4 g. Lungs were similarly fixed in Bouin's solution followed by storage in formalin. In both experiments, the 5 lung lobes of each animal were coded and examined.

Macroscopically visible metastases were counted using a dissecting microscope. In the absence of macroscopic lung metastases, lung lobes were embedded in paraffin. 4 histological sections at least 20 µm apart were cut through each lobe and stained with haematoxylin and eosin. The rationale for cutting 4 sections is based on work by Thrall et al (1997), who showed in tumour biopsies that, for quantification of hypoxic marker labelling, 4 randomly selected sections provide an accurate estimate of the truly labelled area. The presence of microscopic metastasis was evaluated at a 10× magnification using a transmitted light microscope. Lungs were classified as positive if at least one section revealed a micrometastasis. Likewise, lungs were scored as negative in the absence of any micrometastases.

### Data evaluation

Hypoxic fractions, defined as the percentage of $pO_2$ values lower than 5 mm Hg, and median $pO_2$ values were computed from the histogram, calculated from the pooled needle track readings of each individual tumour, using the $pO_2$ pool software package (Eppendorf). A Mann–Whitney test was applied to test differences in oxygenation between the KHT-C and SCC-VII tumours (Figure 1A, B) and differences in number of macroscopic lung metastases

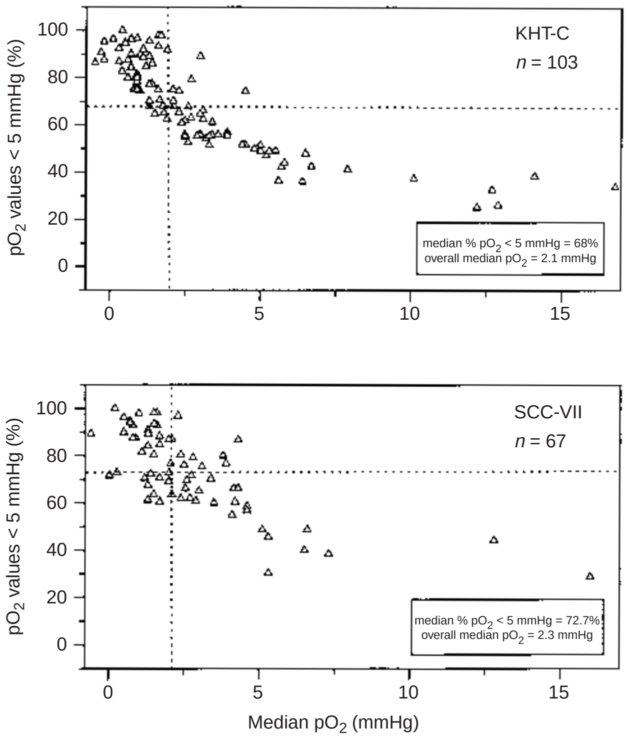

**Figure 1** Percentage of $pO_2$ values lower than 5 mmHg as a function of median $pO_2$ for (**A**) 103 KHT-C and (**B**) 67 SCC-VII tumours, measured at a tumour weight of 0.3–0.4 g. Each point represents the measurements from an individual tumour. The dashed lines indicate the overall median value of each parameter for the group of KHT-C tumours (**A**) and SCC-VII tumours (**B**)

*© 2001 Cancer Research Campaign*

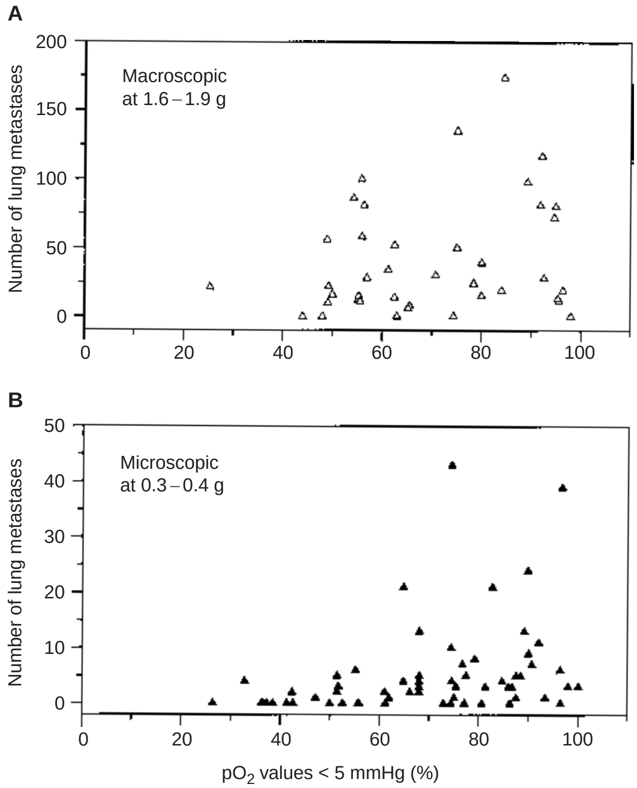

**Figure 2** Number of lung metastases in each of 40 (**A**) or 63 (**B**) mice bearing KHT-C tumours as a function of the percentage of $pO_2$ values <5 mmHg measured at tumour weight 0.3–0.4 g. The mice were killed for assessment of macroscopic lung metastases when the tumours reached a weight of 1.6–1.9 g (**A**) and for assessment of microscopic lung metastases at tumour weight of 0.3–0.4 g (**B**)

between hypoxic and non-hypoxic KHT-C tumours. A Spearman rank correlation coefficient was calculated for evaluation of the correlation between macroscopic lung metastases and oxygen status in the primary KHT-C tumours (Figure 2). Pearson's Chi-squared test with Yates correction was applied to compare frequencies in the contingency tables. The level of significance was defined as $P < 0.05$ (two-sided).

### RESULTS

### Tumour oxygenation measurements

The results of the oxygen measurements in mouse tumours of 0.3–0.4 g are plotted in Figure 1A for 103 individual KHT-C tumours and in Figure 1B for 67 individual SCC-VII tumours. In both figures, the percentage of $pO_2$ values lower than 5 mmHg is plotted as a function of the median $pO_2$. The dashed lines indicate the median value for each parameter. The hypoxic proportion, represented by the percentage of $pO_2$ values lower than 5 mmHg ranges from 25.3% to 100% (median 68%) and from 28.6% to 100% (median 72.7%) in KHT-C and SCC-VII respectively. There is no statistically significant difference in median hypoxic proportion between the two tumour cell lines ($P = 0.52$). For both tumour cell lines, a considerable inter-tumour heterogeneity in oxygenation is observed. The spectra of inter-tumour heterogeneity however are similar for both tumour cell types.

### Metastasis assessment

*Macroscopic lung metastasis at tumour weight 1.6–1.9 g*

For KHT-C, the number of macroscopically visible lung metastases as counted using a dissecting microscope is plotted versus the fraction of $pO_2$ values lower than 5 mmHg at a tumour weight of 0.3–0.4 g in Figure 2A. This graph is updated from previously reported preliminary results (De Jaeger et al, 1998). Although there seems to be a trend suggesting increasing incidence of lung metastases with increasing hypoxic fraction, there is only a weak, non-significant correlation ($r_s = 0.19$, $P = 0.25$). Also, analysis of these data by dividing the tumours at the median value for the hypoxic fraction demonstrated that the number of lung metastases is not significantly different for primary tumours with an oxygenation level above or below the median (median number of lung metastases 18.5 versus 29, $P = 0.21$). Macroscopic lung metastases were not detected in any of the 46 SCC-VII tumours analysed.

*Microscopic lung metastasis at tumour weight 0.3–0.4 g*

Because all but 2 of the animals with KHT-C tumours examined at a primary tumour weight of 1.6–1.9 g had metastases and many had a large number of metastases, we also examined the extent of metastases at an earlier stage of tumour growth.

Table 1 summarizes the results of the evaluation of microscopic lung metastasis for KHT-C when the tumour-bearing animals were assessed for hypoxic fraction and then killed at a tumour weight of 0.3–0.4 g. Each individual animal was classified in this 2 × 2 table according to whether the hypoxic fraction in the tumour (at tumour weight 0.3–0.4 g) was above/equal to or below the overall median percentage of values lower than 5 mmHg, and whether it was positive or negative for lung metastases, based on the evaluation of 4 independent histological sections. In 45 of 63 lungs, at least one microscopic metastasis was present. In 32 of these 45 mice with lung metastases, measurement of oxygen level in the primary revealed a hypoxic proportion above or equal to 68%, representing the overall median percentage of values < 5 mmHg. Thus, hypoxic tumours seem to metastasize at an earlier stage of growth more frequently as compared to better-oxygenated tumours. Likewise, a higher proportion of negative lungs was observed in mice with relatively better-oxygenated tumours. These proportions are significantly different ($\pm\chi^2 = 6.178$, $P = 0.0143$). The number of micrometastases observed in the lungs from each mouse is plotted versus the fraction of $pO_2$ values lower than 5 mmHg in Figure 2B for comparison with the data for macroscopic metastases. Analysis of these results demonstrated a significant correlation between the hypoxic proportion and the number of microscopic lung metastases ($r_s = 0.50$, $P < 0.01$). The number of lung metastases is also significantly different for primary tumours with a hypoxic proportion above or below the median of 68% (median number of lung metastases 3.5 versus 1.0, $P < 0.01$).

**Table 1** Classification of 63 animals with KHT-C tumours according to % $pO_2$ values <5 mmHg ≥ or < the overall median of 68%, and positive or negative for microscopic lung metastasis. Oxygen measurements and lung metastases were both evaluated at tumour weight 0.3–0.4 g

| % $pO_2$ values | Lung metastasis | | Total |
|---|---|---|---|
| <5 mmHg | Positive | Negative | |
| ≥68 | 32 | 6 | 38 |
| <68 | 13 | 12 | 25 |
| Total | 45 | 18 | 63 |

*© 2001 Cancer Research Campaign*

A similar series of studies was undertaken with SCC-VII tumours. Table 2a shows that the incidence of spontaneous metastasis at 0.3–0.4 g is very low in this tumour cell line with detectable metastasis development in only 3/21 animals. The numbers are very small and do not suggest any correlation between oxygenation status and metastasis formation in SCC-VII. Following the observation of low metastatic incidence at tumour weight 0.3–0.4 g in SCC-VII, we also examined the lungs from mice killed at a primary tumour weight 1.6–1.9 g for microscopic metastasis. These results are summarized in Table 2b. Even at a larger tumour size, only 6/46 tumours demonstrated detectable lung metastasis. Again, there was no correlation with the oxygenation status of the primary tumour at 0.3–0.4 g.

### DISCUSSION

In the present study the effect of hypoxia in vivo on the formation of distant metastases was examined in 2 murine tumour cell lines. We utilized the Eppendorf Histograph to measure oxygen concentrations because it is currently the only clinically applicable technique whose strong predictive value in terms of radioresistance, tumour progression and metastasis has been extensively documented in patients (Gatenby et al, 1988; Höckel et al, 1993; Brizel et al, 1996; Höckel et al, 1996; Nordsmark et al, 1996; Brizel et al, 1997; Fyles et al, 1998; Sundfør et al, 1998, Knocke et al, 1999, Lyng et al, 2000).

Data on the relationship between direct measurements of oxygenation in rodent tumours and radiation curability have been reported (Nordsmark et al, 1995) but the relationship to metastasis formation has, to our knowledge, not been previously addressed in a murine model.

We observed substantial intra-tumour heterogeneity in oxygenation of KHT-C and SCC-VII tumours. Both tumour types, measured at tumour weight 0.3–0.4 g show variation within an almost identical range (Figure 1A, B). This range is in agreement with our preliminary data (De Jaeger et al, 1998) and with data obtained in our lab in a different group of KHT-C tumours (Kavanagh et al, 1999). We have previously shown in KHT tumours that consecutive measurements of $pO_2$ in the same tumour give similar results and that there is a correlation in individual KHT tumours between measurements of $pO_2$ made when the tumours were 0.3–0.4 g and when they were 1.0–1.1 g in size (De Jaeger et al, 1998). We and others have postulated that variation in $pO_2$ values, measured in individual tumours from the same cell line, at the same size and transplanted in identical hosts, are likely to be a consequence of differences associated with local tumour growth and stochastic development of vasculature rather than intrinsic genetic differences (Rockwell and Moulder, 1990; De Jaeger et al, 1998). Similar heterogeneity in oxygenation, but over a wider range has been reported in human tumours (Gatenby et al, 1988; Höckel et al, 1993, 1996; Brizel et al, 1995, 1996, 1997; Nordsmark et al, 1996; Fyles et al, 1998; Sundfør et al, 1998).

The SCC-VII and KHT-C tumours were found to be quite dissimilar in terms of metastasis formation, despite the comparable oxygenation status in vivo of both tumour types. For SCC-VII, the overall incidence of lung metastases was very low. There was no difference whether the lungs were examined at a tumour weight 0.3–0.4 g or 1.6–1.9 g, either for macroscopic or microscopic metastases. Also, there was no correlation with oxygenation status in the primary tumour. However, when pooling the results of Tables 2a and 2b a slight trend for a relationship between metastasis formation and oxygenation status was observed (7/9 mice with lung metastasis had hypoxic primary tumours) but the correlation was not significant.

In the KHT-C model metastases were much more frequent. At a tumour weight 1.6–1.9 g, macroscopic metastases were present in the majority of the lungs with only 2/40 mice not showing macroscopically visible pulmonary metastases. As depicted in Figure 2A only a weak non-significant correlation was observed between oxygenation of the primary tumour at tumour weight 0.3–0.4 g and the number of lung metastases observed at weight 1.6–1.9 g. We hypothesized that a possible relationship could be obscured by the presence of massive lung metastases when the primary tumours reach a weight of 1.6–1.9 g and by the fact that the tumours become increasingly hypoxic as their weight increases above 0.3 g (De Jaeger et al, 1998). In a previous study (Hill et al, 1986) we showed that KHT-C fibrosarcoma starts seeding metastases into the lungs at a tumour weight of 0.25 g (corresponding leg diameter = 7.5–8 mm).

Consequently, we decided to examine whether hypoxia in the primary tumour correlates with metastasis formation in the lungs at an earlier time point (tumour weight 0.3–0.4 g) in the process of seeding. Table 1 and Figure 2b represent the results for 63 KHT-C tumours and illustrate that hypoxic KHT-C tumours, defined as tumours with a percentage of $pO_2$ readings < 5 mmHg above or equal to the median percentage of 68%, gave rise to significantly more positive lungs and micrometastases as compared to better-oxygenated tumours. This result, suggesting that hypoxic KHT tumours are more likely to be metastatic is consistent with the clinical data obtained in head and neck cancer, cervix cancer and soft tissue sarcoma (Brizel et al, 1996; Höckel et al, 1996; Rofstad et al, 2000; Walenta et al, 2000). Preliminary analysis from the clinical study of oxygenation of cervix cancer being conducted at the Princess Margaret Hospital (Fyles et al, 1998) has indicated increased nodal metastases in the patients with more hypoxic tumours (Pitson et al, 2001).

There is growing evidence from laboratory studies supporting the clinical observations that the significance of hypoxia in cancer

**Table 2a** Classification of 21 animals with SCC- VII tumours according to % $pO_2$ values <5 mmHg ≥ or < the overall median of 72.7%, and positive or negative score for microscopic lung metastasis. Oxygen measurements and lung metastases were both evaluated at tumour weight 0.3–0.4 g

| % $pO_2$ values <5 mmHg | Lung metastasis Positive | Lung metastasis Negative | Total |
|---|---|---|---|
| ≥72.7 | 2 | 8 | 10 |
| <72.7 | 1 | 10 | 11 |
| Total | 3 | 18 | 21 |

**Table 2b** Classification of 46 animals with SCC- VII tumours according to % $pO_2$ values <5 mmHg ≥ or < the overall median of 72.7%, and positive or negative score for microscopic lung metastasis. Oxygen measurements were performed at tumour weight 0.3–0.4 g. Lung metastases were evaluated at tumour weight 1.6–1.9 g

| % $pO_2$ values <5 mmHg | Lung metastasis Positive | Lung metastasis Negative | Total |
|---|---|---|---|
| ≥72.7 | 5 | 19 | 24 |
| <72.7 | 1 | 21 | 22 |
| Total | 6 | 40 | 46 |

© 2001 Cancer Research Campaign may extend far beyond the traditional scope of radioresistance. Young et al (1988) have shown that exposure of murine KHT fibrosarcoma cells to hypoxia in vitro results in a transient enhancement of their ability to form lung metastases. They suggested that gene amplification, associated with DNA over-replication, was responsible for the enhanced metastatic potential. Further studies of the expression of a number of metastasis-related genes following hypoxic exposure did not identify a gene whose altered expression correlated with the increased metastatic potential of the cells, although vascular endothelial growth factor (VEGF) was up-regulated (Jang and Hill, 1997). In similar experiments with human melanoma cells, other groups found that exposure to hypoxia promotes metastasis formation (Hartmann et al, 1999; Rofstad and Danielsen, 1999). They demonstrated a correlative up-regulation of the expression of VEGF (Hartmann et al, 1999; Rofstad and Danielsen, 1999) and angiogenin (Hartmann et al, 1999), both potent angiogenic factors. The effect of the tumour microenvironment on metastasis has been reviewed recently (Rofstad, 2000).

Reynolds et al (1996) studied the impact of fluctuating hypoxia on the frequency of mutations arising in a shuttle vector carried in a tumorigenic mouse cell line. They detected a 3–4-fold increase in mutation frequency under severe hypoxic conditions. Their results indicate that the environmental conditions within solid tumours can be mutagenic and suggest that hypoxia mediates tumour progression by induction of genetic instability. Similar findings have been reported by Sandhu et al (2000), who examined HPRT mutations in MN-11 tumor cells. Graeber and colleagues (Graeber et al, 1996; Kim et al, 1997) found that hypoxia can mediate the selection of cells deficient in genes of normal regulatory pathways. This group demonstrated that hypoxia provides a selective pressure for cells mutant in the *p53* tumour-suppressor gene resulting in decreased apoptotic potential and establishment of a more malignant phenotype. Furthermore, it has been well documented that hypoxic stimuli can alter the expression of a myriad of genes, transcription factors, growth factors, cytokines, metabolic and invasive enzymes (Stoler et al, 1992; Dachs and Stratford, 1996; Jang and Hill, 1997; Sutherland, 1998; Graham et al, 1999; Dachs and Tozer, 2000; Semenza, 2000). Despite tremendous progress in understanding fundamental mechanisms of hypoxia-induced genetic, metabolic and chemical alterations in cells, it still remains unclear how and whether these alterations act in concert. Also, oxygenation is not a binary physiological condition and the contribution of transient and chronic changes on these interactions remains to be determined. The results in this paper establish the KHT fibrosarcoma as a model system for such studies.

Moreover, it should be pointed out that hypoxia is not the only tumour microenvironmental condition affecting tumour progression. Other factors, such as pH and low glucose may play a role (Schlappack et al, 1991). Also, hypoxia per se does not necessarily imply metastatic ability. This is clearly illustrated in the SCC-VII model where, despite severe hypoxia in the primary tumours, cells fail to metastasize. Limitations related to the model, such as heterotopic implantation could have contributed to the metastatic inefficiency of SCC-VII cells. It is well known from the work of Fidler (1990) that orthotopic models give rise to higher metastatic rates. Therefore, the i.m. transplanted KHT model is likely to be a more relevant representative of natural tumour behaviour than i.m. transplanted SCC-VII tumours.

In summary, this is the first report investigating the relationship between direct measurements of tumour oxygenation in vivo and metastatic behaviour of rodent tumours. In the KHT-C model, early metastatic ability was found to be enhanced in hypoxic tumours. The present results are consistent with previous clinical and laboratory findings indicating that hypoxia may contribute to malignant progression. The availability of this model allows in vivo testing of hypoxia-directed strategies leading to potentially effective treatment. However, as demonstrated in the SCC-VII model, there are factors other than hypoxia which affect the metastatic ability of tumour cells.

### ACKNOWLEDGEMENTS

This work was supported by a grant from the National Cancer Institute of Canada with funds raised by the Terry Fox Run. R Kuba is acknowledged for technical assistance. KDJ was a Research Fellow of the Department of Radiation Oncology, University of Toronto. KDJ's current address: The Netherlands Cancer Institute, Amsterdam, The Netherlands.

### REFERENCES

Bristow RG and Hill RP (1998) Molecular and Cellular Basis of Radiotherapy. In: *The Basic Science of Oncology*, Tannock IF and Hill RP (eds) pp 309–313. McGraw-Hill: New York

Bristow RG, Hardy PA and Hill RP (1990) Comparison between in vitro radiosensitivity and in vivo radioresponse of murine tumor cell lines. I: Parameters of *in vitro* radiosensitivity and endogenous cellular glutathione levels. *Int J Radiat Oncol Biol Phys* **18**: 133–145

Brizel DM, Rosner GL, Prosnitz LR and Dewhirst MW (1995) Patterns and variability of tumor oxygenation in human soft tissue sarcomas, cervical carcinomas, and lymph node metastases. *Int J Radiat Biol* **32**: 1121–1125

Brizel DM, Scully SP, Harrelson JM, Layfield LJ, Bean JM, Prosnitz LR and Dewhirst MW (1996) Tumor oxygenation predicts for the likelihood of distant metastases in human soft tissue sarcoma. *Cancer Res* **56**: 941–943

Brizel DM, Sibley GS, Prosnitz LR, Scher RL and Dewhirst MW (1997) Tumor hypoxia adversely affects the prognosis of carcinoma of the head and neck. *Int J Radiat Oncol Biol Phys* **38**: 285–289

Dachs GU and Stratford IJ (1996) The molecular response of mammalian cells to hypoxia and the potential for exploitation in cancer therapy. *Br J Cancer-Supplement* **74**: S126–S132

Dachs GU and Tozer GM (2000) Hypoxia modulated gene expression: angiogenesis, metastasis and therapeutic exploitation. *Eur J Cancer* **36**: 1649–1660

De Jaeger K, Merlo F, Kavanagh MC, Fyles AW, Hedley D and Hill RP (1998) Heterogeneity of tumor oxygenation: relationship to tumor necrosis, tumor size, and metastasis. *Int J Radiat Oncol Biol Phys* **42**: 717–721

Fidler IJ (1990) Critical factors in the biology of human cancer metastasis: twenty-eighth G.H.A. Clowes memorial award lecture. *Cancer Res* **50**: 6130–6138

Fyles AW, Milosevic M, Wong R, Kavanagh MC, Pintilie M, Sun A, Chapman W, Levin W, Manchul L, Keane TJ and Hill RP (1998) Oxygenation predicts radiation response and survival in patients with cervix cancer. *Radiother Oncol* **48**: 149–156

Gatenby RA, Kessler HB, Rosenblum JS, Coia LR, Moldofsky PJ, Hartz WH and Broder GJ (1988) Oxygen distribution in squamous cell carcinoma metastases and its relationship to outcome of radiation therapy. *Int J Radiat Oncol Biol Phys* **14**: 831–838

Graeber TG, Osmanian C, Jacks T, Housman DE, Koch CJ, Lowe SW and Giaccia AJ (1996) Hypoxia-mediated selection of cells with diminished apoptotic potential in solid tumours. *Nature* **379**: 88–91

Graham CH, Forsdike J, Fitzgerald CJ and Macdonald-Goodfellow S (1999) Hypoxia-mediated stimulation of carcinoma cell invasiveness via upregulation of urokinase receptor expression. *Int J Cancer* **80**: 617–623

Hartmann A, Kunz M, Kostlin S, Gillitzer R, Toksoy A, Brocker EB and Klein CE (1999) Hypoxia-induced up-regulation of angiogenin in human malignant melanoma. *Cancer Res* **59**: 1578–1583

Hill RP, Young SD and Ling V (1986) Metastatic cell phenotypes: Quantitative studies using the experimental metastasis assay. *Cancer Rev* **5**: 118–151

 *© 2001 Cancer Research Campaign*

Höckel M, Knoop C, Schlenger K, Vorndran B, Baussmann E, Mitze M, Knapstein PG and Vaupel P (1993) Intratumoral $pO_2$ predicts survival in advanced cancer of the uterine cervix. *Radiother Oncol* **26**: 45–50

Höckel M, Schlenger K, Aral B, Mitze M, Schaffer U and Vaupel P (1996) Association between tumor hypoxia and malignant progression in advanced cancer of the uterine cervix. *Cancer Res* **56**: 4509–4515

Ikeda E, Achen MG, Breier G and Risau W (1995) Hypoxia-induced transcriptional activation and increased mRNA stability of vascular endothelial growth factor in C6 glioma cells. *J Biol Chem* **270**: 19761–19766

Iyer NV, Kotch LE, Agani F, Leung SW, Laughner E, Wenger RH, Gassmann M, Gearhart JD, Lawler AM, Yu AY and Semenza GL (1998) Cellular and developmental control of $O_2$ homeostasis by hypoxia-inducible factor 1 alpha. *Genes Dev* **12**: 149–162

Jang A and Hill RP (1997) An examination of the effects of hypoxia, acidosis, and glucose starvation on the expression of metastasis-associated genes in murine tumor cells. *Clin Exp Metastasis* **15**: 469–483

Kavanagh MC, Sun A, Hu Q and Hill RP (1996) Comparing techniques of measuring tumor hypoxia in different murine tumors: Eppendorf $pO_2$ Histograph, [3H]misonidazole binding and paired survival assay. *Radiat Res* **145**: 491–500

Kavanagh MC, Tsang V, Chow S, Koch CJ, Hedley D, Minkin S and Hill RP (1999) A comparison in individual murine tumors of techniques for measuring oxygen levels. *Int J Radiat Oncol Biol Phys* **44**: 1137–1146

Kim CY, Tsai MH, Osmanian C, Graeber TG, Lee JE, Giffard RG, DiPaolo JA, Peehl DM and Giaccia AJ (1997) Selection of human cervical epithelial cells that possess reduced apoptotic potential to low-oxygen conditions. *Cancer Res* **57**: 4200–4204

Knocke TH, Weitmann HD, Feldmann HJ, Selzer E and Potter R (1999) Intratumoral pO2-measurements as predictive assay in the treatment of carcinoma of the uterine cervix. *Radiother Oncol* **53**: 99–104

Lyng H, Sundfor K, Trope C and Rofstad EK. (2000) Disease control of uterine cervical cancer: relationship to tumor oxygen tension, vascular density, cell density and frequency of mitosis and apoptosis measured before treatment and during radiotherapy. *Clin Cancer Res* **6**: 1104–1112

Nordsmark M, Horsman MR, Overgaard M, Bentzen SM and Overgaard J (1995) The predictive value of oxygen electrode measurements to radiotherapy simulation in an experimental tumor model. *Radiation Research Society Annual Meeting: San Jose, California*. Abstract # P21–311

Nordsmark M, Overgaard M and Overgaard J (1996) Pretreatment oxygenation predicts radiation response in advanced squamous cell carcinoma of the head and neck. *Radiother Oncol* **41**: 31–39

Pitson G, Fyles AW, Milosevic M, Syed A and Hill RP (2001) Tumor size and oxygenation are independent predictors of nodal disease in patients with cervix cancer. *Int J Radiet Oncol Biol Phys* (submitted).

Reynolds TY, Rockwell S and Glazer PM (1996) Genetic instability induced by the tumor microenvironment. *Cancer Res* **56**: 5754–5757

Rockwell S and Moulder JE (1990) Hypoxic fractions of human tumors xenografted into mice: a review. *Int J Radiat Oncol Biol Phys* **19**: 197–202

Rockwell S, Moulder JE and Martin DF (1986) Effectiveness and biological effects of techniques used to induce hypoxia in solid tumors. *Radiother Oncol* **5**: 311–319

Rofstad EK (2000) Microenvironment-induced cancer metastasis. *Int J Radiat Biol* **76**: 589–605

Rofstad EK and Danielsen T (1999) Hypoxia-induced metastasis of human melanoma cells: involvement of vascular endothelial growth factor-mediated angiogenesis. *Br J Cancer* **80**: 1697–1707

Rofstad EK, Sundfor K, Lyng H and Trope CG. (2000) Hypoxia-induced treatment failure in advanced squamous cell carcinoma of the uterine cervix is primarily due to hypoxia-induced radiation resistance rather than hypoxia-induced metastasis. *Br J Cancer* **83**: 354–359

Sandhu JK, Haggani AS and Birnbolm HC (2000) Effect of dietary vitamin E on spontaneous or nitric oxide donor-induced mutations in a mouse tumor model. *J Natl Cancer Inst* **92**: 1429–1433

Schlappack OK, Zimmermann A and Hill RP (1991) Glucose starvation and acidosis: effect on experimental metastatic potential, DNA content and MTX resistance of murine tumour cells. *Br J Cancer* **64**: 663–670

Semenza GL (2000) Hypoxia, clonal selection, and the role of HIF-1 in tumor progression. *Crit Rev Biochem Mol Biol* **35**: 71–103

Stoler DL, Anderson GR, Russo CA, Spina AM and Beerman TA (1992) Anoxia-inducible endonuclease activity as a potential basis of the genomic instability of cancer cells. *Cancer Res* **52**: 4372–4378

Sundfør, Lyng H and Rofstad EK (1998) Tumour hypoxia and vascular density as predictors of metastasis in squamous cell carcinoma of the uterine cervix. *Br J Cancer* **78**: 822–827

Sutherland RM (1998) Tumor hypoxia and gene expression – implication for malignant progression and therapy. *Acta Oncol* **37**: 567–574

Thrall DE, Rosner GL, Azuma C, McEntee MC and Raleigh JA (1997) Hypoxia marker labeling in tumor biopsies: quantification of labeling variation and criteria for biopsy sectioning. *Radiother Oncol* **44**: 171–176

Walenta S, Wetterling M, Lehrke M, Schwickert G, Sundfor K, Rofstad EK and Mueller-Kleiser W (2000) High lactate levels predict likelihood of metastases, tumor recurrence and restricted patient survival in human cervical cancers. *Cancer Res* **60**: 916–921

Young SD, Marshall RS and Hill RP (1988) Hypoxia induces DNA overreplication and enhances metastatic potential of murine tumor cells. *Proc Natl Acad Sci USA* **85**: 9533–9537

*© 2001 Cancer Research Campaign*
